# Eco-Friendly Procedure for Rendering the Antibacterial and Antioxidant of Cotton Fabrics *via* Phyto-Synthesized AgNPs With *Malva sylvestris* (MS) Natural Colorant

**DOI:** 10.3389/fbioe.2021.814374

**Published:** 2022-01-12

**Authors:** Mousa Sadeghi-Kiakhani, Ali Reza Tehrani-Bagha, Fateme Sadat Miri, Elaheh Hashemi, Mahdi Safi

**Affiliations:** ^1^ Department of Organic Colorants, Institute for Color Science and Technology, Tehran, Iran; ^2^ Department of Bioproducts and Biosystems, Aalto University, Espoo, Finland; ^3^ Department of Chemistry, Faculty of Sciences, Shahid Rajaee Teacher Training University, Tehran, Iran; ^4^ Department of Color Physics, Institute for Color, Science and Technology, Tehran, Iran

**Keywords:** antimicrobial, cotton fabric, natural colorant, Malva sylvestris, antioxidant

## Abstract

There is a growing interest for producing multifunctional cellulose fabrics using green and sustainable technology. In this study, we explored an eco-friendly procedure for dyeing cotton fabrics with *Malva sylvestris* (MS) as a natural colorant and rendering antibacterial cotton fabric by the silver nanoparticles. MS colorants were extracted from dried petals in water using the ultrasound technique, cotton fabrics were dyed with the extracted MS colorant at 100°C for 90 min. The colorimetric data and colorfastness properties were investigated in the absence and presence of tannic acid (TA) as a bio-mordant. Results indicated that MS dye had a high potential for reducing the silver nitrate, so that the silver particle size distribution on cotton fabric was obtained 50–80 nm, and TA had a positive effect on the MS extract and reduced Ag on the cotton. Furthermore, the reduction of bacterial growth of the dyed cotton considerably (up to 99%) improved by AgNPs. The wash-, and light-fastness properties of samples dyed with MS were enhanced from moderate to good-very good by mordanting.

## 1 Introduction

The preparation of silver nanoparticles (AgNPs) with natural reducing agents has attracted the attention of researchers, due to the possibility to shorten the synthesis procedure (Veeraputhiran, 2013; Kelkawi et al., 2016). It has been reported that several plants have potential to reduce silver nitrate in nanoparticle dimensions, and also phyto-synthesized AgNPs have been currently offered as operative method with promising results to replace synthetic chemical pesticides with unsafe effects to non-target species (Rout et al., 2012; Singh et al., 2012).

*Malva sylvestris* (MS) is attractive flower spikes from the family of Malva-Mallows with five-petaled rose-purple color. The active ingredients include mucilage, tannins, malvin, malvidin, oenine, and delphinidin (Razavi et al., 2011). The proximate composition including fatty acid, mineral content, total flavonoids and mucilage were measured and reported in the literatures (Yeole et al., 2010). The presence of the mentioned compounds and phytochemicals like terpenoids, flavones, and flavonoids in this plant provides excellent antibacterial and antioxidant activities (Esteves et al., 2009; Jeambey et al., 2009; Tabaraki et al., 2012).

Cotton is a cellulosic fiber that has attracted much attention among the different types of fibers, because it is natural, biodegradable, and sustainable (Jayaramudu et al., 2010; Sedighi et al., 2014; Gopinath et al., 2016; Binoj et al., 2016) and have different hydroxyl groups (Ren et al., 2017; Bu et al., 2019). Currently, several procedures have been reported for the production of antibacterial cotton fibers with AgNPs. Zhu et al. using NaBH_4_ as a reducing agent synthesized AgNPs on cotton fibers (Zhu et al., 2009). They suggested that the average and particle size distribution of AgNPs depend on the concentration of reducing agent. In another study, researchers used different reducing agents like sodium citrate, ascorbate and ethylene glycol with a microwave technique in the synthesis of AgNPs on the textiles (de Santa Maria et al., 2009; Yue et al., 2019). During the synthesis of AgNPs on the textiles, the use of unsafe reducing agents is destructive for the nature, which it imposes largely practical limitations in the field of biology. Hence, the green reducing agents such as natural colorants, ascorbic acid and glucose were suggested and used for the synthesis of AgNPs (Antony et al., 2011). The shortcoming of aforementioned reducing agents is the production of AgNPs with larger particle sizes and thus lower antibacterial activities (Li et al., 2011a). For example, ascorbic acid as a reducing agent used for the synthesis of silver particles (250 nm) in the cellulose matrix with relatively poor distribution (Li et al., 2011b). Natural colorants can be employed as reducing agents for the synthesis of Ag NPs with smaller size with lower environmental impact (Antony et al., 2011; Gong et al., 2019). For example, Aladpoosh et al. reported the preparation of AgNPs on the cotton using by ashes of *S. Rosmarinus* (Aladpoosh et al., 2014). The excellent antibacterial properties in low content of Ag were provided by negligible variation in tensile strength and colorimetric data of samples. Sri Ramkumar et al. synthesized AgNPs by reduction with the prepared seaweed extract (Ramkumar et al., 2017). The shape of Ag nano structures was spherical with size ranges between 4 and 24 nm and the synthesized AgNPs reduced significantly the growth of bacterial and fungal. Therefore, the challenge is that the synthesis of nano size AgNPs with uniform particle size distribution *via* natural colorants and green procedures.

In this study, the antioxidant and antibacterial cotton fabric was produced through the phyto-synthesize of AgNPs using extracted MS colorants as an economical and ecologically satisfactory green process. This novel affordable method can be developed by utilizing natural resources to produce antibacterial and antioxidant cotton fabrics. The objectives were to 1) the extraction of colorants from MS flowers, 2) use the extracted colorants for dyeing of cotton fabrics pre-mordanted with tannic acid (TA), 3) synthesis of AgNPs by MS extract as a natural reducing agent on the cotton, and 4) investigate the colorimetric data, colorfastness, antioxidant and antibacterial properties of colored samples.

## 2 Experimental

### 2.1 Materials and Analytical Instruments

Purple petals of MS flowers were purchased from a local market. Commercially bleached cotton fabric with specification of plain weave, weft and warp/cm: 36 and 32 was provided by Savalan Co., Iran. Tannic acid (purity >95%), silver nitrate (AgNO_3_) (purity: 99%) and metal mordants including copper (II) sulfate, aluminum (III) sulfate, and ferrous (II) sulfate heptahydrate supplied by Merck, Germany, and all of them were on an analytical scale.

The absorbance of extracted MS dye in solutions was recorded by Cecil 9200 double beam UV-Vis spectrophotometer. The solvent for the dye extraction and dilution before the measurements was distilled water. The crystalline structure of AgNPs on the cotton fabrics was determined by an EQuniox 3000, INEL X-ray diffractometer (XRD). The FTIR spectra of the samples were measured by Nicolet Nexus 670 Fourier Transform Infrared instrument in the range of 4,000–400 cm^−1^ using KBr disks. The morphology of cotton samples and the elemental composition was measured by Scanning Electron Microscopy -Energy Dispersive (SEM-EDS) Spectroscopy. A Color eye 7000A, X-rite reflectance spectrophotometer with illuminant D65 and 10° standard observer was employed to obtain the reflectance and colorimetric data.

### 2.2 Extraction of Natural Colorants From MS

**FIGURE 1:**
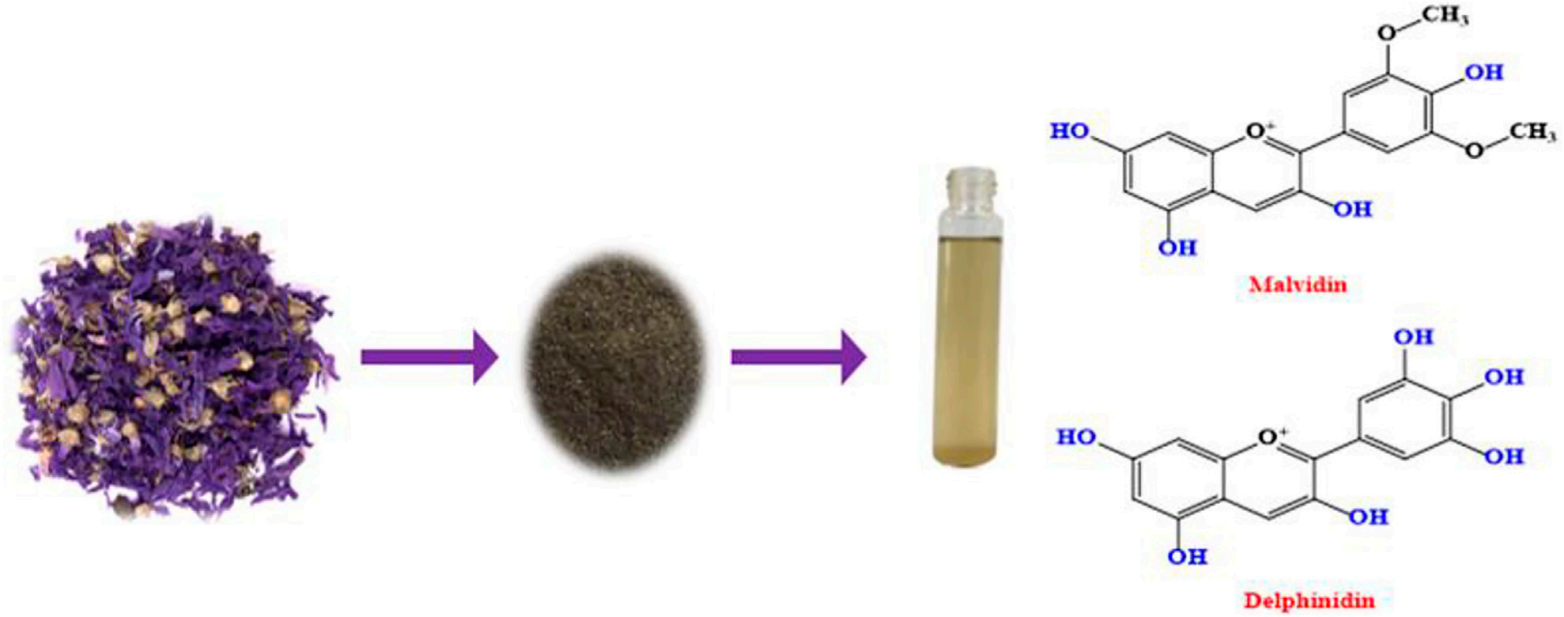
The preparation of MS extract.

The MS petals dried at 40°C, and milled with 60 mesh granulometry. The extraction of natural colorants from MS powder was performed by distilled water. The initial concentration of MS powder in water was in the range of 5–20 g/L. The extraction bath boiled for 2 h and then filtration was performed using a Whatman filter paper. The extracted colorants were separated from water by evaporation and drying at 40°C. The preparation of MS powder and the chemical structures of the extracted colorants from MS petals is shown in Figure 1.

### 2.3 Preparation of AgNPs

The stock solution of AgNO_3_ (50 mM) was prepared in deionized water at room temperature. The AgNO_3_ ions was reduced into AgNPs by MS colorant as a green reducing agent. The color change of the extracts and reduced Ag ions within few hours was measured by UV-Visible spectrophotometer. To obtain the stable colloidal AgNPs with proper shape and size, significant factors were considered for the optimization of the process including the initial concentration of MS colorant, silver nitrate concentration, temperature, and time.

### 2.4 Mordanting and Dyeing Procedures

TA was applied on the cotton fabrics (1 g) at various concentrations 1–20% (o.w.f.), temperature 80°C, time 60 min and a liquor ratio of 40:1. The treated samples were squeezed off and air-dried. Before the dyeing process, the samples were also mordanted with metals salts (i.e., alum 5% o.w.f., copper sulfate 2% o.w.f., and ferrous sulfate 2% o.w.f.).

Dyeing of cotton fabrics was done by Smart dyer rapid dyeing apparatus. The temperature and duration of dyeing process were 100°C and 90 min, respectively. Finally, the dyed fabrics were then rinsed, washed, squeezed and dried.

### 2.5 Application of AgNPs and MS Extract on the Cotton Fabric

Cotton fabrics (1 g) treated by different amounts of AgNO_3_ (0.1, 0.5, 1 and 2% o.w.f) and MS extracted colorant (50% o.w.f.). The pH and L.R of the solution were neutral and 40:1, respectively. Temperature increased up to boiling and the process lasted for an hour. After treatment, all samples were washed and dried.

### 2.6 Color Measurements

The color strength (K/S) value of dyed samples has been measured by a reflectance spectrophotometer, and determined using Kubelka-Munk equation (Eq. 1) (Shabbir et al., 2019):

$$\frac{K}{S}=\frac{{\left(1-R\right)}^{2}}{2R}-\frac{{\left(1-R_{0}\right)}^{2}}{2R_{0}}$$ (1)

where K is the absorption coefficient, and S is the scattering coefficient. R and R_0_ are the reflectance of the cotton fabric after and before the dyeing process, respectively. Also, the color difference (ΔE) of dyed samples was calculated by Eq. 2 (Shabbir et al., 2019).

$$\Delta E=\sqrt{{\left(\Delta L^{*}\right)}^{2}+{\left(\Delta a^{*}\right)}^{2}+{\left(\Delta b^{*}\right)}^{2}}$$ (2)

### 2.7 Colorfastness Testing

The colorfastness to wash of dyed samples was measured by ISO standard methods including ISO 105-C06. Moreover, the light fastness measurement was determined by ISO 105-B02. It is noteworthy that the wash fastness of the samples was evaluated from 1: weak to 5: excellent using the gray scales. The colorfastness to light of dyed samples was also evaluated using the blue scales from 1: weak to 8: excellent.

### 2.8 Free Radical Scavenging Test

Di(phenyl)-(2,4,6-trinitrophenyl)iminoazanium (DPPH) was used to determine the antioxidant percent of the treated samples with AgNPs reduced with MS extract (Lisanti et al., 2016). For this purpose, DPPH (0.15 mM) was added in methanol (40 ml) and cotton samples (2.5 cm^2^) was immersed in the solution. The reaction was performed in dark condition for 30 min. The absorbance performance and color change of solutions, and antioxidant activity of dyed samples was calculated using Eq. 3 (Lisanti et al., 2016).

$$\mathrm{Antioxidant}\left(\%\right)=\frac{C-D}{C}\times 100$$ (3)

Where, D and C are the absorbance of the treated and untreated cotton samples at λ_max._ = 517 nm, respectively.

### 2.9 Antibacterial Testing

The antibacterial property of treated samples with AgNPs reduced by MS extract was measured against *S. aureus* and *E. coli* bacteria. A loopful of each bacteria (10 µL) was added in a culture medium (10 ml). Then, 100 µL of culture medium containing of each bacteria was added to a sterilized petri dish including 20 ml nutrient agar (Mueller-Hinton agar). The incubation of all samples was performed at 37°C for 24 h and the bacterial reduction percent was evaluated by Eq. 4 (Chen et al., 2019).

$$\mathrm{Bacterial}\;\mathrm{reduction}\left(\%\right)=\frac{\left(B-A\right)}{B}\times 100$$ (4)

Where, A and B represent the surviving bacterial cells on the plates inoculated with a bacterial solution resulting from the dyed and raw cotton fabrics, respectively.

## 3 Results and Discussion

### 3.1 Extraction

**FIGURE 2:**
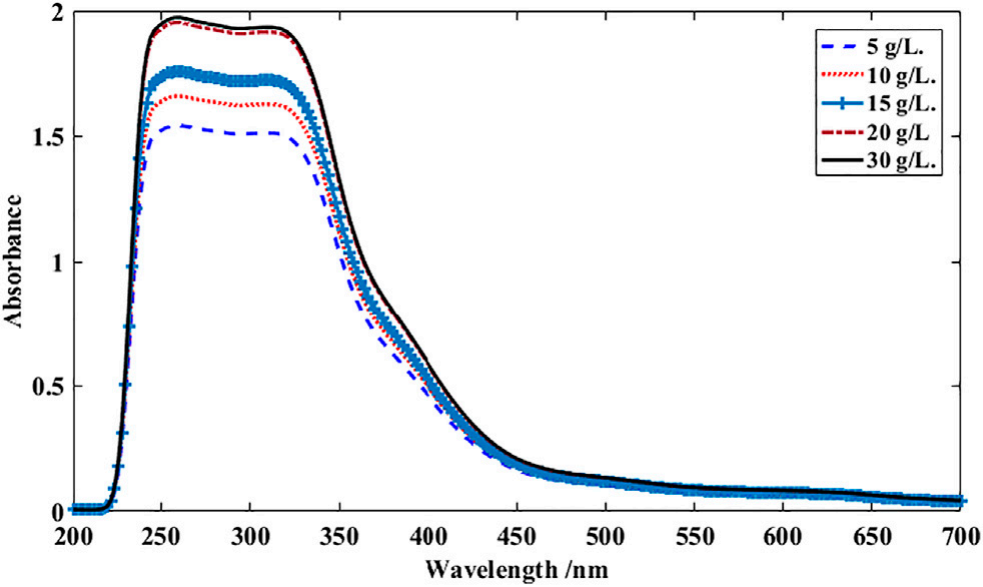
UV-Vis spectra dye solutions at various initial concentration of MS powder. MS powder was dispersed in distilled water and boiled for 2 h.

The absorbance behavior of extracted colorants from MS petals at various initial concentrations is shown in Figure 2. The absorbance of the extracted solutions increased by the initial concentration of MS powdered petals up to 20 g/L (i.e., saturation concentration). The further increase of the MS powder to 30 g/L did not enhance the extraction efficiency. The more concentration of MS powder in water, the more colorants can be solubilized and extracted. The MS colorants have a certain solubility limit in water which has been reached by adding 20 g/L of the MS powdered petals to the extraction bath (Safapour et al., 2018).

The presence of phenolic compounds in the MS colorants provide a pale yellow color with a wavelength of maximum absorbance 220–350 nm (Lattanzio et al., 2008; Silva et al., 2020). The wavelength absorption bands between 250 and 290 nm can be related to phenolic compounds while flavones and flavonols can be appeared around 250 and 350 nm (Mahmoodi Esfanddarani et al., 2017).

**FIGURE 3:**
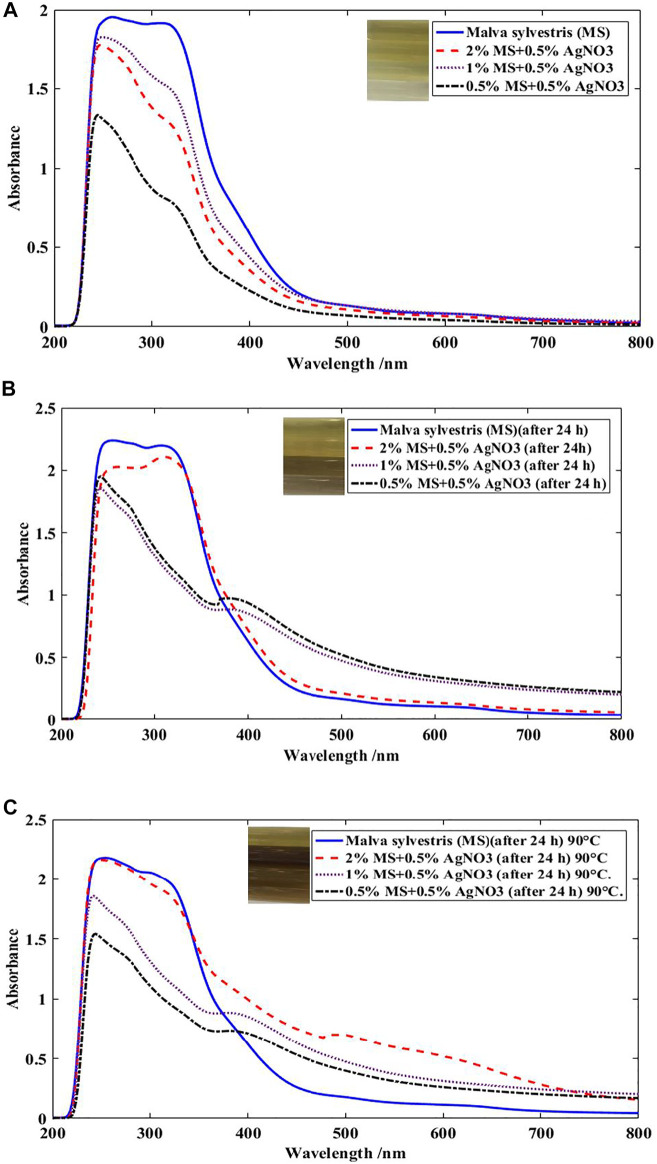
UV-Visible spectra of MS extract at various concentrations in the presence of 0.5% Ag ions **(A)** at room temperature after 2–3 min, **(B)** at ambient temperature after 24 h, **(C)** at ambient temperature after 24 h followed by heating at boiling for 1 h

The excitation of surface Plasmon resonance (SPR) stated as a reason for change the color of the solution containing MS extract and AgNPs. This phenomena strongly specifies the active compounds in MS extract can reduce silver particles acceptably and biosilver was formed (Mahmoodi Esfanddarani et al., 2017). Also, the reduction of silver particles and alteration of Ag^+^→Ag^0^ was confirmed by the absorption in *λ* at around 400–430 nm (Figure 3A) (Govindarajan and Benelli, 2016). Measurements of the absorption spectra of the solution including silver particles and MS extract at different times (24 h) showed that the Ag NPs could be stable, so that the particle size was acceptable and aggregation of the particles was negligible. The synthesized AgNPs in solution were stable for at least 24 h and no sediment particles are formed and the solution is clear (Figure 3B). The perfect reduction of the silver nitrate by MS extract was identified after 24 h, and consequently the surface plasmon peak was observed at 480 nm (Ren et al., 2017). Furthermore, the increasing of temperature (90°C) of solution including MS extract and Ag particles verified the stability of the AgNPs by heating at boiling for 1 h (Figure 3C).

**FIGURE 4:**
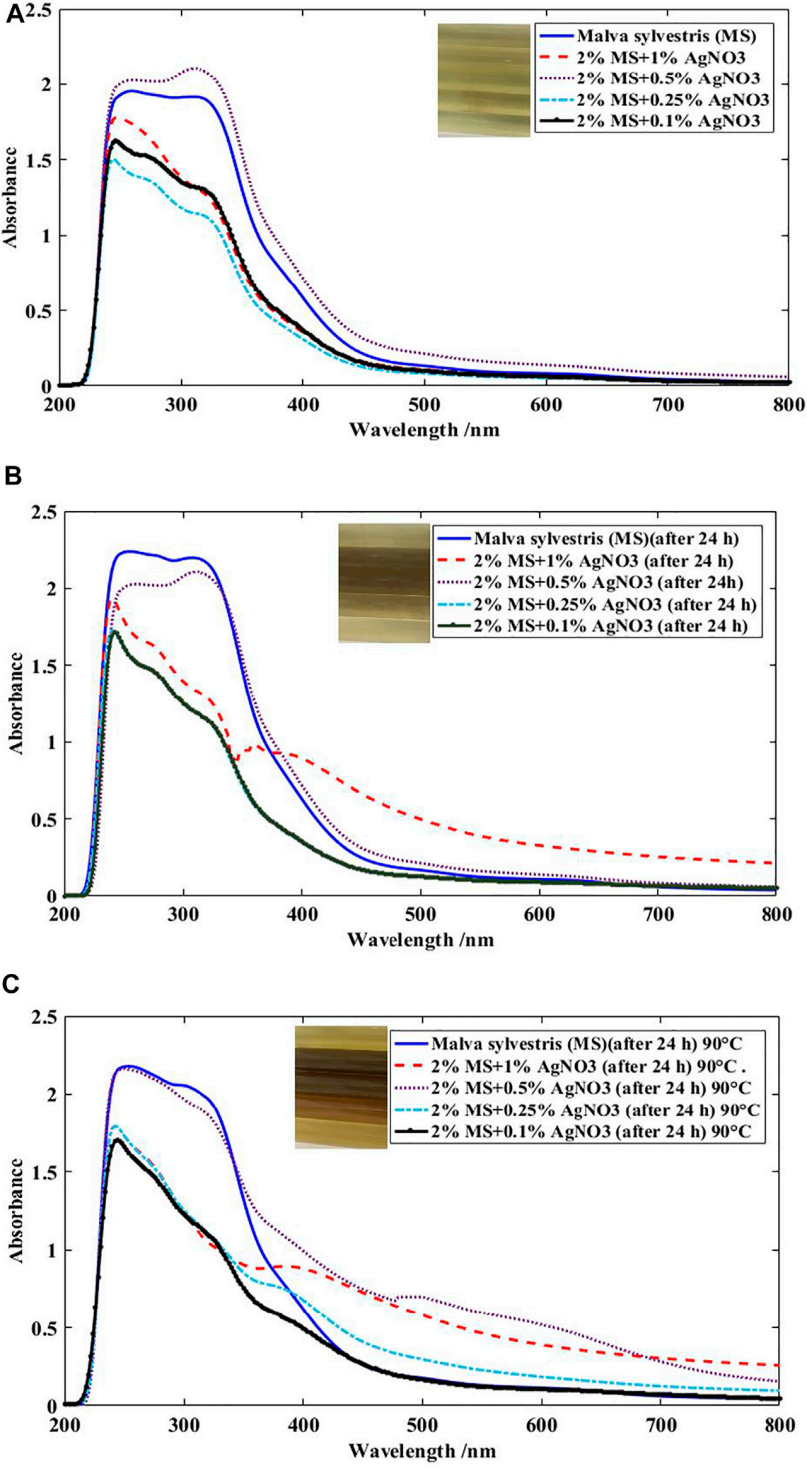
UV-Visible spectra of 2% MS extract at various amounts of Ag **(A)** at room temperature after 2–3 min, **(B)** at ambient temperature after 24 h, **(C)** at ambient temperature after 24 h followed by heating at boiling for 1 h

Different initial concentrations of silver nitrate were investigated in the inherent reduction property of phenols and phenolic acids extracted from MS. Figure 4 shows the absorption spectra of the solution in a constant amount of MS extract, different amounts of AgNO_3_ under different times of reaction. Results indicated that Ag NPs were formed in the more concentrations of silver in the solution, which it is observable *via* a new peak appeared at 417 nm (Govindarajan and Benelli, 2016; Mahmoodi Esfanddarani et al., 2017). Moreover, the synthesis of Ag NPs is influenced by time and amount of silver. So that, the characteristics of the UV-visible spectra were different at different times and silver values. It was observable that the *λ*_max_ values of solutions were at around 417 nm at 0.5% Ag ion. So, it can be concluded that the preparation of AgNPs could be performed through the reduction by MS extract at the Ag ion value of 0.5–1%. A wide peak with low absorption in the range of 450–515 nm at 0.5% amount of Ag ions at 90°C is visible, which indicates that the size of the silver particles formed is larger than the nanoscale and the particle size distribution of Ag NPs is inhomogeneous in the solution (Govindarajan and Benelli, 2016; Mahmoodi Esfanddarani et al., 2017). So, the concentration of silver in the solution was a main factor to phyto-synthesis of Ag NPs.

**FIGURE 5:**
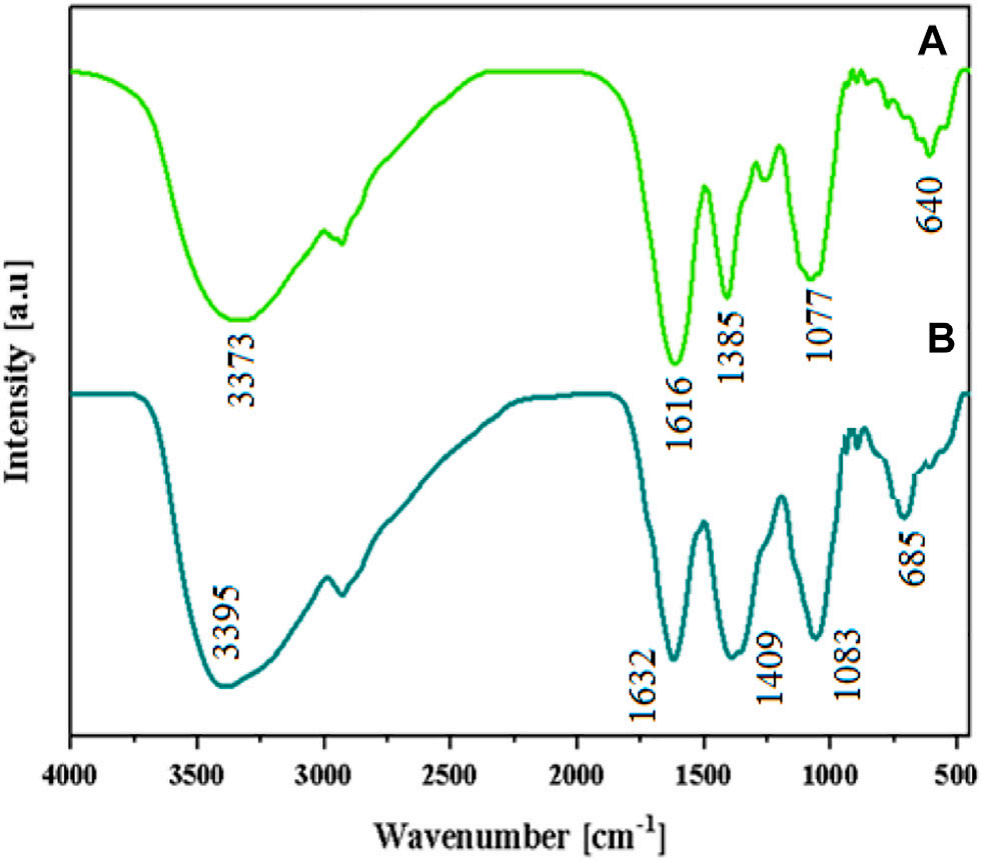
FTIR spectra of the extracted colorant in the **(A)** absence and **(B)** presence of AgNPs.

The FTIR spectra of dried MS extract and silver nanoparticles are shown in Figure 5. The diverse functional groups of flavonoids and tannins are detectable with intense and relatively broadband at 3373 (-OH), 1616 (-C=O), 1385 (-C=C-), 1077 (-C-O-C-), and 640 cm^−1^ (-C-C-) (Oo et al., 2009; Ricci et al., 2015). Similarly, in the mixture of MS/AgNPs, the intense, sharp band at 1632 cm^−1^ and 1409 cm^−1^ is related to a carbonyl groups and the C=C vibration of aromatic rings, respectively. (Oo et al., 2009). Also, C-O stretching appeared at 1083 cm^−1^ is associated to cellulose and hemicellulose linkages in the MS extract (Oo et al., 2009; Ricci et al., 2015). The peaks in the 685 region can also be related to –C-C-, -C-N-, and –C=O in the MS extract (Liu et al., 2008; Oo et al., 2009). The bond formation between silver nanoparticles and the MS dye is observable at below 1000 cm^−1^. The shift in the above mentioned peaks after reaction with AgNO_3_ with MS dyes signifying that -OH, -C=O and -NH-CO groups on the surface of natural MS dye.

### 3.2 Characterization of Cotton Fabrics

**FIGURE 6:**
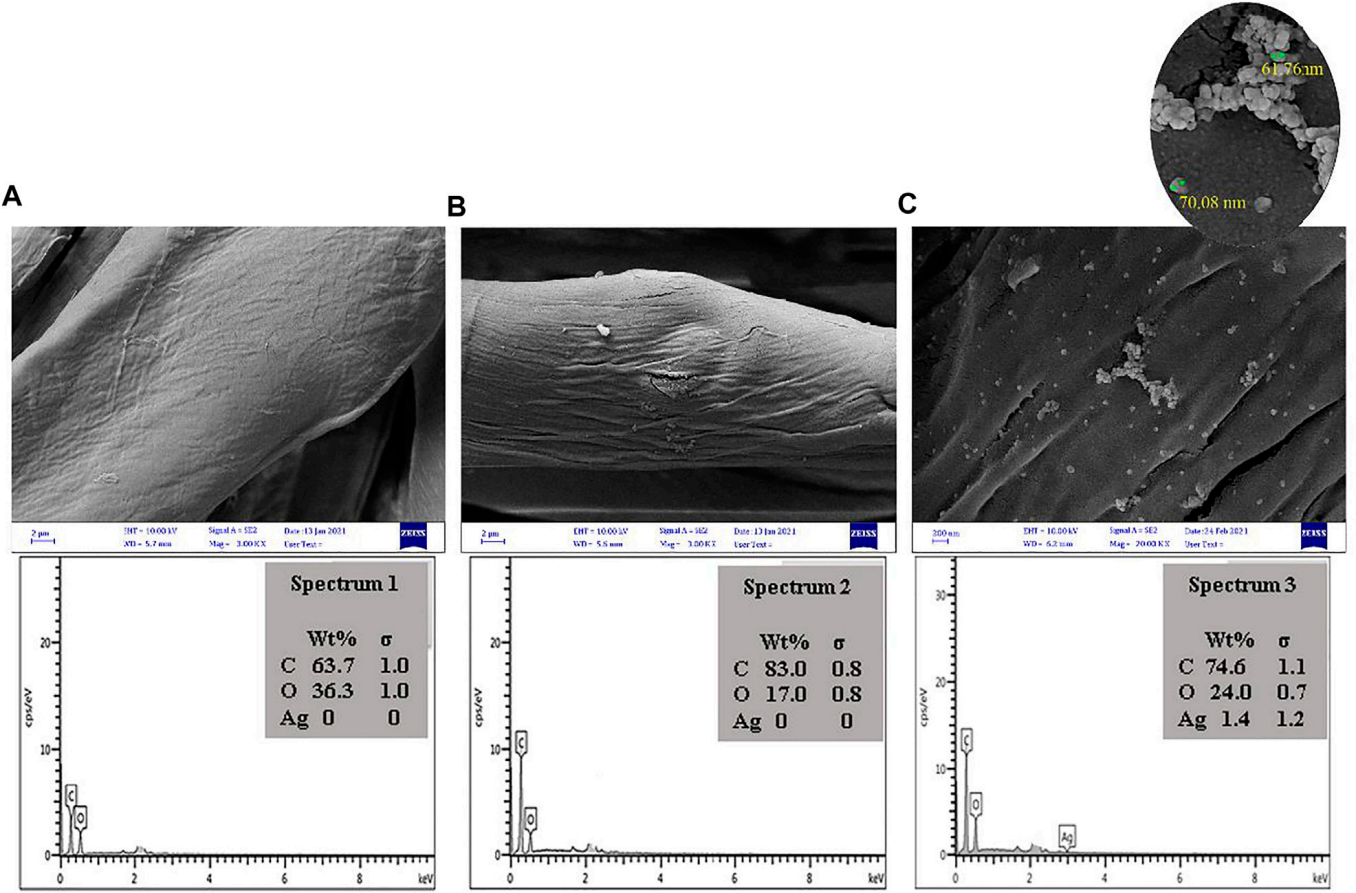
SEM photographs of **(A)** cotton fabrics, **(B)** cotton fabric treated by 10%o.w.f. TA and **(C)** treated cotton fabric by 10% o.w.f. TA and 5%%o.w.f. Ag after application of natural dye.

The presence AgNPs and their particle size distribution on the cotton fabrics was investigated by SEM analysis (Figure 6). Based on the SEM image analyses, the surface properties of cotton fabric had changed after treatment with MS and AgNPs, and the presence of AgNPs on the cotton fabrics was verified by a typical peak at 3 keV. Results showed that the average particle size of AgNPs was around 50–80 nm (Zhou et al., 2017; Shabbir et al., 2019).

**FIGURE 7:**
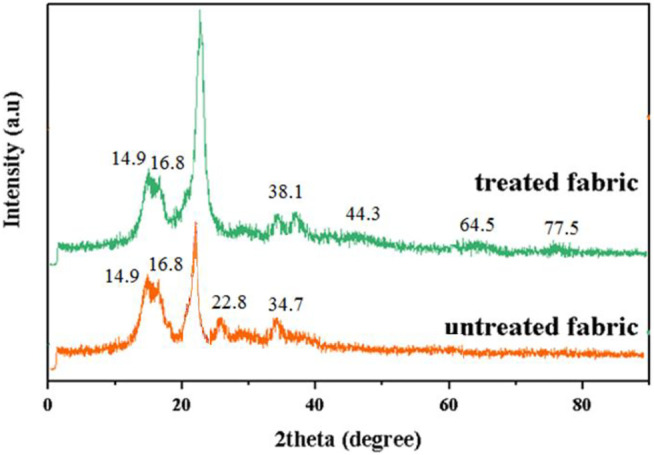
X-ray diffraction (XRD) analysis of cotton and treated cotton with MS + 10% TA + 1%AgNPs).

The grafting of silver nanoparticles on cotton fabric was also investigated using X-ray instrument (Figure 7). The high-intensity peaks at 2θ = 14.9°, 16.8°, 22.8°, and 34.7° indicated that crystalline structure of cellulose in cotton fibers (Lu and Shen, 2011). Also, the strong peaks at 2θ = 38.1° (major), 44.3°, 64.5°, and 77.5° was observable after treatment of cotton fabric with MS extract and Ag ions. The data confirmed the diffraction from the (111), (200), (220), and (311) planes of silver with face-centered cubic structure, respectively. The average crystal size of the AgNPs on the cotton fabric was calculated by *Scherrer* equation Eq. 4. Results showed the D amount for AgNPs was obtained around 70 nm (Liu et al., 2010):

$$D=\frac{K\times \lambda }{\beta \times \mathrm{Cos}\left(\theta \right)}$$

where D is the average crystal size, K is the *Scherrer* coefficient (0.9), *k* is the X-ray wavelength (0.154 nm), *β* is the full width at half-maximum in radians and θ is the Bragg angle (Liu et al., 2010).

### 3.3 Dyeing Properties

#### 3.3.1 Effect of the Tannic Acid on the Dye Absorption

Tannic acid (TA) was applied as a bio-mordant to enhance the dye adsorption and fixation rate, and the colorimetric data of dyed samples was given in Table 1. The color strength (K/S) and color difference (∆E) values for the dyed cotton sample were relatively low which was improved slightly for the samples pre-mordanted with TA. This can be described by the more hydrogen bonding between cotton fabrics treated by TA and the phenolic compounds identified in MS colorants (Prabhu and Teli, 2014). However, the K/S values reported in Table 1 are relatively low which shows the low affinity of MS colorants towards cellulose. Also, the amounts of a*, b*and hue of dyed samples is constant after mordanting with TA.

**TABLE 1:** Effect of TA concentration on the colorimetric data of dyed samples.

Treatments	L*	a*	b*	ΔE	C*	h	K/S
MS	80.94	3.16	1.81	—	3.64	29.79	1.26
MS+5% TA	78.29	5.37	1.06	3.52	6.39	11.16	1.68
MS+10% TA	77.89	3.33	4.38	3.90	5.50	52.8	1.76
MS+20% TA	78.08	2.55	2.85	3.10	7.20	54.51	1.46

#### 3.3.2 Effect of Metal Mordant on the Dye Absorption

**SCHEME 1:**
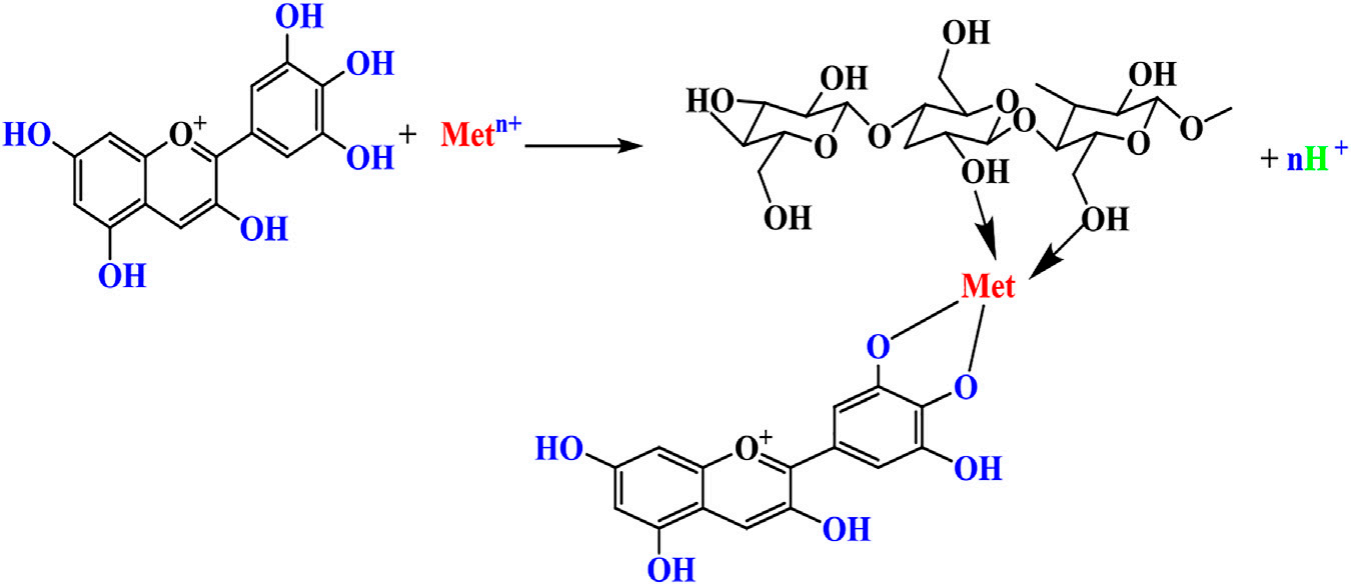
Metal ions can bridge natural colorants to cellulose polymer chains. Met: Al, Fe, Cu, Sn metal ions

Due to the weak interactions between cotton and natural colorants, metal salts are often used as mordants to bridge the colorants with cellulose chains. The mechanism is typically based on the complex formation and coordination as illustrated in Scheme 1. The metal ions can be aluminum (Al^3+^), iron (Fe^2+^), or copper (Cu^2+^) for linking MS colorants to cellulose polymer chains in cotton (Mussak and Bechtold, 2009).

Table 2 represents the dyeing characteristics of samples pre-mordanted with various metal salts (e.g., Al^3+^, Fe^2+^, Cu^2+^, Sn^2+^) at boiling temperature in pH 7. The K/S values of samples mordanted with iron and copper salts were increased significantly while the samples mordanted with aluminum and tin salts had a slight increase in color strength. Here, the presence of a metal mordant could enhance the color strength of cotton samples due to the formation new bonding between insoluble metal with hydroxyl group of tannin phenolic on the substrate, and consequently the fixation rate on the cotton fabric was improved (Mussak and Bechtold, 2009).

**TABLE 2:** Colorimetric data of the cotton fabric with and without mordanting.

Samples	L*	a*	b*	C*	K/S
MS	80.94	3.16	1.81	3.64	1.26
MS+3%Al	82.13	4.54	−0.45	4.56	1.45
MS+3%Fe	67.32	2.04	3.68	4.21	2.47
MS+3%Cu	74.66	2.56	7.20	7.64	1.83
MS+3%Sn	83.64	2.83	−0.12	2.83	1.57
MS+10% Tannic acid (TA)	77.89	3.33	4.38	5.50	1.76
MS+10% TA + 3%Al	77.53	3.03	4.12	5.12	1.70
MS+10% TA + 3%Fe	44.74	2.74	9.53	9.92	4.47
MS+10% TA + 3%Cu	60.34	3.22	14.86	15.20	3.86
MS+10% TA + 3%Sn	76.82	2.68	−1.28	2.96	2.33

The cotton fabrics pre-mordanted with aluminum salt showed the lowest amount of dye absorption, due to aluminum ions (Al^3+^) cannot bridge MS colorants with hydroxyl groups of cellulose polymer chains in cotton as efficiently as other metals salts including Fe^2+^ and Cu^2+^. This may be related to the electron configuration of aluminum and its valence electronic structure.

We can also notice the change in the chroma (C*) values of the dyed samples pre-mordanted with various salts. The changes in chroma were observed in the cases of Fe and Cu mordants. So that, the chroma of color on dyed samples noticeably improved and accordingly the lightness of samples considerably decreased. It can be ascribed to the inherent nature of the Fe and Cu mordants and the providing of dark shades on the cotton fabrics with MS natural dye extract (Mussak and Bechtold, 2009).

Table 3 indicates the color coordinates of the samples mordanted with TA and dyed with MS extract/AgNPs. The *in-situ* formation of AgNPs verified to be an efficient mordanting method and the absorption of MS dye on the samples were almost doubled. However, the color brightness of the mordanted samples (L* values) was lower compared with the lightness of non-treated dyed samples with L* = 80.94 (Li et al., 2015).

**TABLE 3:** Colorimetric analysis of samples with and without AgNPs (MS 50% o.w.f.).

Samples	L*	a*	b*	C*	h	K/S
MS	83.64	2.83	0.12	2.83	2.42	1.26
MS+0.25% AgNPs	71.29	4.17	12.16	12.85	71.09	2.39
MS+0.5%AgNPs	69.78	4.02	13.08	13.68	72.93	2.26
MS+1%AgNPs	64.57	3.44	5.68	6.64	58.79	2.39
MS+10% TA	80.94	3.16	1.81	3.64	29.79	1.76
MS+10% TA + 0.25% AgNPs	70.08	6.12	8.46	10.44	54.12	2.28
MS+10% TA + 0.5%AgNPs	64.62	6.58	12.03	13.71	61.31	2.65
MS+10% TA + 1%AgNPs	60.50	4.32	7.30	8.49	59.37	2.91

### 3.4 Colorfastness Properties

The colorfastness of the dyed samples against washing, light and rubbing was investigated, and ratings are shown in Table 4. The mordanted cotton fabrics by TA had considerably good fastness rates. The fastness to light of the samples was just moderate which can be explained by low photo-stability of MS colorants. The light fastness values of the mordanted cotton fabrics are enhanced in the presence of TA and AgNPs. The use of Al and Sn mordants leads to more fading than Fe and Cu, which can be attributed to the effect of mordants type on the fastness properties of natural dyes (Kiumarsi and Gashti, 2015). The chemical structure and intermolecular interaction of dye constitutes in MS maybe protect against photo fading and degradation (Kiumarsi and Gashti, 2015).

**TABLE 4:** Fastness ratings of the dyed cotton fabrics with and without mordants.

Samples	Washing fastness			Light fastness
	SC*	SN*	CC	
MS	5	5	3–4	5
MS+3%Al	5	5	4–5	4
MS+3%Fe	5	5	3	4–5
MS+3%Cu	5	5	4–5	5
MS+3%Sn	5	5	4	4
MS+10% Tannic acid (TA)	5	5	3–4	5–6
MS+10% TA + 3%Al	5	5	3–4	5
MS+10% TA + 3%Fe	5	5	4–5	4
MS+10% TA + 3%Cu	5	5	4–5	4–5
MS+10% TA + 3%Sn	5	5	5	4

Table 5 shows the impact of Ag NPs on the colorfastness characteristics of the dyed samples. Due to the creation of insoluble color complexes on the treated cotton fabrics, the rating of fastness to wash, in terms of color change, increase up to 1/2–1 grade when TA and Ag NPs were used. The colorfastness to light of cotton samples slightly improved and we discussed this in the previous section.

**TABLE 5:** Fastness ratings of the dyed cotton fabrics with and without AgNPs (MS 50% o.w.f.).

Samples	Washing fastness			Light fastness
	SC*	SN*	CC	
MS	5	5	3–4	5
MS + 0.25% AgNPs	5	5	3	5
MS + 0.5%AgNPs	5	5	3	5
MS + 1%AgNPs	5	5	3	4
MS+10% Tannic acid (TA)	5	5	3–4	5–6
MS+10% TA + 0.25% AgNPs	5	5	4	5–6
MS+10% TA + 0.5%AgNPs	5	5	4	6
MS+10% TA + 1%AgNPs	5	5	3	6

### 3.5 Antibacterial and Antioxidant Activities

Table 6 shows the antibacterial efficiency results of the dyed samples. Data indicated that antibacterial efficiency of the dyed samples with MS colorant without any treatment, with TA and AgNPs were 41–44%. The considerable antibacterial activity of MS colorants can be attributed to the presence of anthocyanin, flavonoids, vitamins, terpenoides (Dulger and Gonuz, 2004; El-Rafie et al., 2010; Shaheen et al., 2021). The antibacterial efficiency of the cotton samples pre-mordanted with TA and dyed with MS colorants was around 80%. The antibacterial efficiency of the dyed samples improved to over 99% in the presence of Ag 1% o.w.f.

**TABLE 6:** Antibacterial activity of cotton samples at various conditions; the concentration of TA and Ag were % owf.

Sample	TA (%owf)	Ag (%owf)	Surviving cells (CFU/ml)		Reduction of bacteria (%)	
			*E. Coli*	*S. aureus*	*E. Coli*	*S. aureus*
Raw cotton	—		7000	7350	—	—
Cotton dyed with MS (50 %o.w.f.)	0	0	3660	3990	44.20	41.92
	10	0	980	1250	82.54	77.40
	10	0.5	24	202	99.30	98.10
	10	1	26	56	99.70	99.60

Hence, it can be inferred that the acceptable antibacterial activities was achieved on the cotton fabrics by 10% TA mordant, MS (50% o.w.f.), and 0.5% or 1% Ag NPs concentration.

The durability of cotton fabrics dyed with MS after 5 and 10 washes was tested and antibacterial activities of each samples was measured (Table 7). The durability of any fabric treatment is an important consideration which should be evaluated. The antibacterial efficiency values were reduced by increasing the number of repeated washing cycles. However, the sample pre-mordanted with TA and covered with AgNPs kept over 94% antibacterial activity even after 10 washes. The antibacterial efficiency for the sample pre-mordanted with TA without any AgNPs was around 62–66% after ten repeated washing cycles. This shows the intrinsic antibacterial properties of MS colorants and TA with polyphenolic chemical structure and their strong intermolecular interactions with cellulose chains of cotton. In general, it was found that the pre-mordanting with TA should be performed in the presence of metal salts for attaining a durable antibacterial activity on the cotton fabrics (Shaheen et al., 2021).

**TABLE 7:** The antibacterial activity of dyed cotton fabrics with MS after 5 and 10 washes.

Sample	TA (%owf)	Ag (%owf)	Reduction (%) (5 repeated washing)		Reduction (%) (10 repeated washing)	
			*E. Coli*	*S. aureus*	*E. Coli*	*S. aureus*
Cotton dyed with MS (50% o.w.f.)	0	0	39.42	41.92	36.60	35.92
	10	0	75.64	77.40	65.85	62.15
	10	0.5	98.20	96.45	95.75	94.05
	10	1	98.40	98.12	96.58	96.36

**FIGURE 8:**
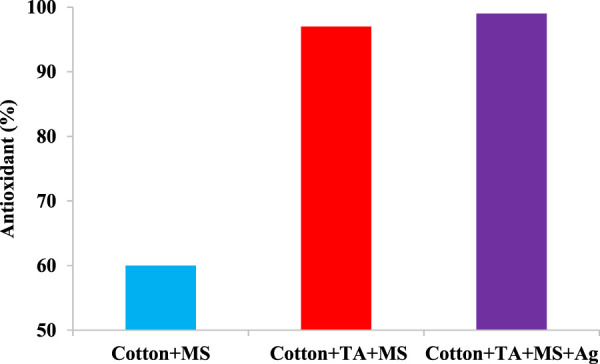
The antioxidant activity of cotton samples dyed with *MS* extracts in presence of TA and AgNPs (MS dye: 2%, t 60 min, T 80°C, Ag 1%).

The existence of phenolic compounds in the MS colorants provides the antioxidant ability (DellaGreca et al., 2009; Cutillo et al., 2006; Gasparetto et al., 2012). The antioxidant potential of all cotton fabrics was measured by the DPPH method (Figure 8). An increase in the percent of antioxidant was observed as the concentration of MS increased up to 50% o.w.f. (DellaGreca et al., 2009; Shaheen et al., 2021), and antioxidant amounts of the cotton sample dyed with MS was 30% lower than of the mordanted with TA. However, the un-mordanted samples dyed with MS enhanced the antioxidant activity by more than 30% (Razavi et al., 2011). In general, the incorporation of TA in the dyeing system not only could provide antibacterial activity on cotton fabrics but also has enhanced the preservation against free radicals. This can also be a reason for the relatively high light-fastness properties of the samples.

## 4 Conclusion

Phyto-synthesis of AgNPs on the cotton fabrics with acceptable antibacterial and antioxidant activities was produced by MS flower. The dye absorption of MS colorants on the cotton fabrics was improved by pre-mordanting with TA. Also, silver ions can increase the color strength of samples other than the dyed sample with MS. Type of mordant was a significant factor on the hue of the colored samples and was different from yellow to brown. The colorfastness ratings of the dyed samples against to wash and light were generally moderate that were enhanced slightly by pre-mordanting. The presence of AgNPs on the dyed samples enhanced their antibacterial efficiency considerably. The antibacterial efficiency of the samples remained over 95% against bacteria for the samples treated with TA and AgNPs even after 10 washes. The textile coloration using natural colorants obtained from MS and *in-situ* coating with AgNPs can be considered as an eco-friendly treatment for cellulose fabrics.

## Data Availability

The original contributions presented in the study are included in the article, further inquiries can be directed to the corresponding author.
